# Photoionization Loss of Mercury's Sodium Exosphere: Seasonal Observations by MESSENGER and the THEMIS Telescope

**DOI:** 10.1029/2021GL092980

**Published:** 2021-04-28

**Authors:** Jamie M. Jasinski, Timothy A. Cassidy, Jim M. Raines, Anna Milillo, Leonardo H. Regoli, Ryan Dewey, James A. Slavin, Valeria Mangano, Neil Murphy

**Affiliations:** ^1^ NASA Jet Propulsion Laboratory California Institute of Technology Pasadena CA USA; ^2^ Laboratory of Atmospheric and Space Sciences University of Colorado Boulder Boulder CO USA; ^3^ Department of Climate and Space Sciences and Engineering University of Michigan Ann Arbor MI USA; ^4^ INAF/IAPS Rome Italy; ^5^ Applied Physics Laboratory John Hopkins University Baltimore MD USA

**Keywords:** exosphere, ions, Mercury, photoionization, plasma, sodium

## Abstract

We present the first investigation and quantification of the photoionization loss process to Mercury's sodium exosphere from spacecraft and ground‐based observations. We analyze plasma and neutral sodium measurements from NASA's MESSENGER spacecraft and the THEMIS telescope. We find that the sodium ion (Na^+^) content and therefore the significance of photoionization varies with Mercury's orbit around the Sun (i.e., true anomaly angle: TAA). Na^+^ production is affected by the neutral sodium solar‐radiation acceleration loss process. More Na^+^ was measured on the inbound leg of Mercury's orbit at 180°–360° TAA because less neutral sodium is lost downtail from radiation acceleration. Calculations using results from observations show that the photoionization loss process removes ∼10^24^ atoms/s from the sodium exosphere (maxima of 4 × 10^24^ atoms/s), showing that modeling efforts underestimate this loss process. This is an important result as it shows that photoionization is a significant loss process and larger than loss from radiation acceleration.

## Key Points

- Photoionization can be a significant loss process to the sodium exosphere with peak loss estimates of 4 × 10^24^ atoms/s
- The photoionization loss process of Mercury's sodium exosphere varies throughout the planet's orbit around the Sun
- More sodium is lost due to photoionization on the inbound leg (true anomaly angle of 180°–360°) of Mercury's orbit than the outbound leg

## 1 Introduction

**Figure 1:**
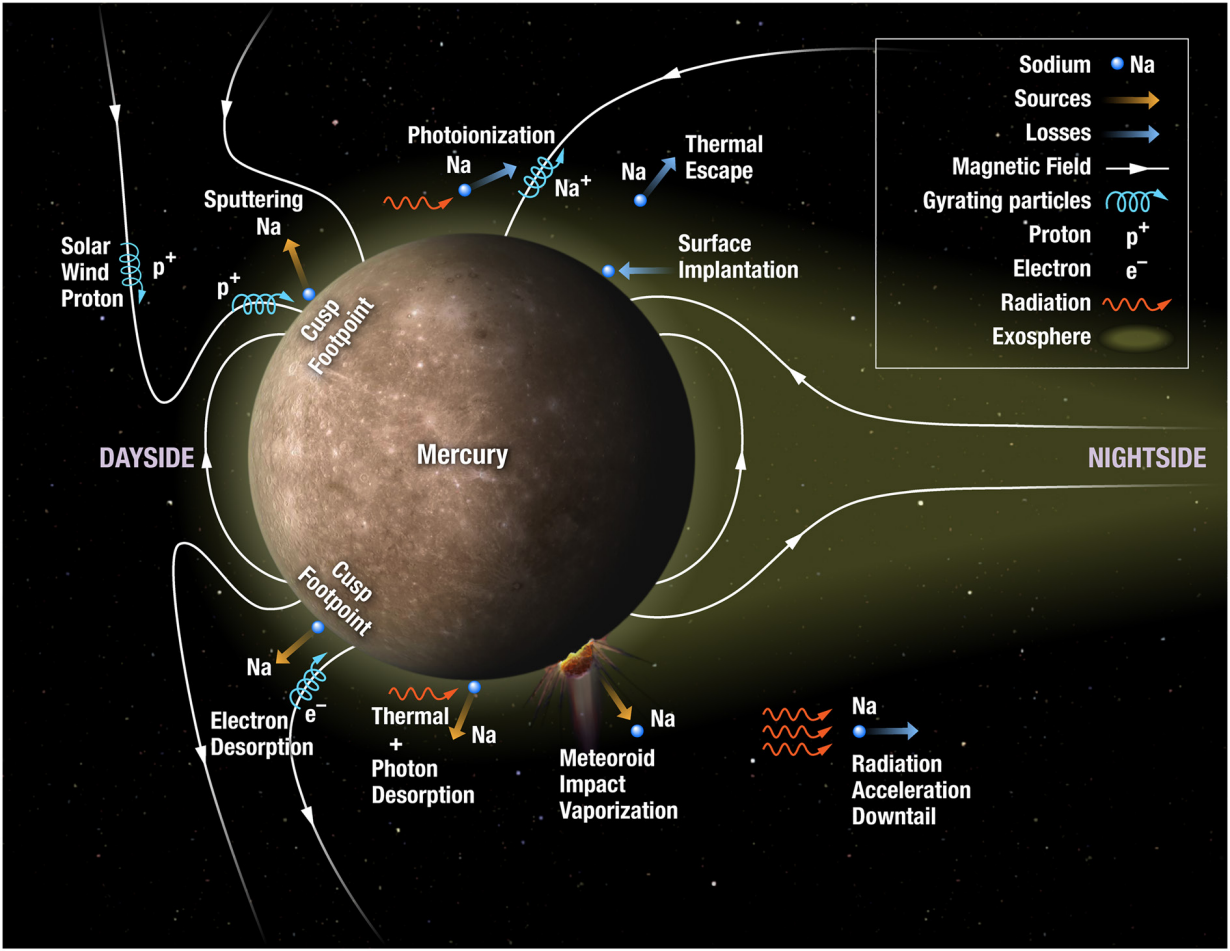
A schematic showing the various exospheric source and loss processes at Mercury.

Mercury has a very tenuous sodium exosphere, where the sodium atoms are more likely to collide with the planetary surface than each other. The neutral sodium exosphere at Mercury is formed from a variety of source and loss processes: Figure 1. Source processes include: photon, thermal, and electron‐stimulated desorption, ion sputtering and impact vaporization. Thermal desorption causes atoms to be released from the surface due to heating (Leblanc & Johnson, 2003; Yakshinskiy & Madey 2000). Photon‐stimulated desorption (PSD) and electron‐stimulated desorption (ESD) are caused by the transfer of an electron to a higher energy state in a sodium atom after the bombardment of photons or electrons, respectively, causing desorption of sodium from the surface (McLain et al., 2011; Yakshinskiy & Madey 2000). PSD dominates Mercury's low latitude dayside exosphere production (Cassidy et al., 2015; Leblanc et al., 2006) and is a function of true anomaly angle, TAA (Mura, 2012; Suzuki et al., 2020). Surface release occurs at Mercury due to the coupling between the solar wind and Mercury's magnetosphere. Magnetic reconnection at the dayside injects plasma into the magnetospheric cusps which can precipitate onto the surface of Mercury (Raines et al., 2014; Slavin et al., 2014, 2019; Sun et al., 2020) and sputter sodium from the surface. Increased sodium emission has been observed during southward interplanetary magnetic field (IMF) orientations, which are most conducive for reconnection (Mangano et al., 2015; Orsini et al., 2018). Large IMF magnitudes are more likely to produce field‐aligned protons which will strike the surface rather than magnetically mirror (Jasinski et al., 2017). Meteroid impact vaporization occurs when a small object strikes the surface of Mercury and causes sodium to be vaporized (Jasinski et al., 2020; Mangano et al., 2007). Micrometeroid impact vaporization is considered another possible major source to the sodium exosphere (Kameda et al., 2009).

Loss processes (Figure 1) remove neutral sodium from the exosphere. Loss processes include: surface implantation, thermal escape, radiation acceleration, and photoionization. Sodium atoms colliding with the surface can be adsorbed (surface implantation). Thermal escape occurs when the thermal energy is sufficiently high (>2.07 eV) to overcome gravitational forces. Radiation acceleration causes sodium to be lost in a cometary‐like tail (Baumgardner et al., 2008; Potter et al., 2002, 2013; Schleicher et al., 2004). This is caused by Mercury's orbit which is the most eccentric in the solar system (eccentricity of 0.2 in comparison to Earth's orbital eccentricity of 0.017).

**Figure 2:**
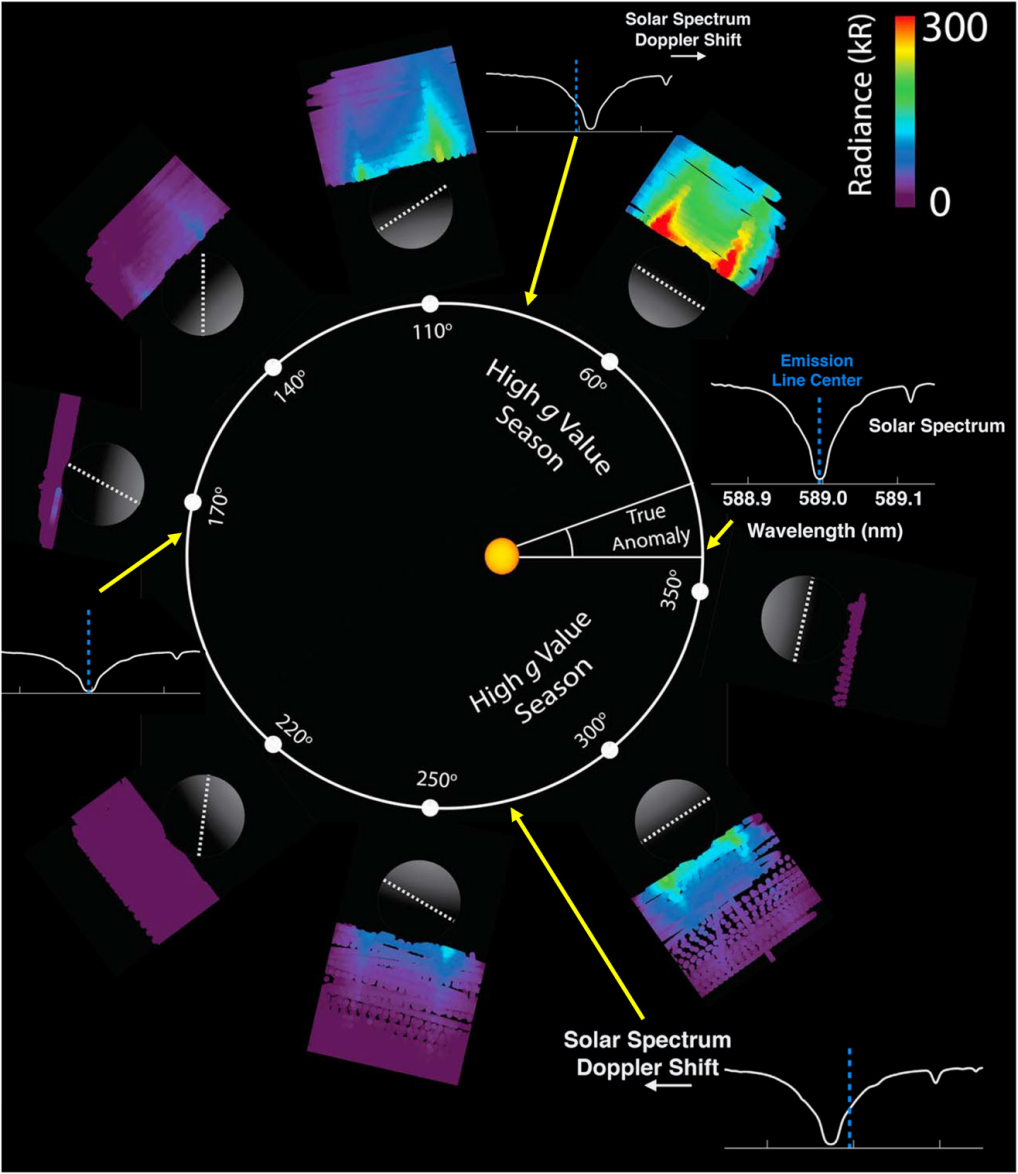
The observed UVVS neutral sodium emission projected onto Mercury's orbital plane, showing the sodium tail which forms during the high *g*‐value portions of Mercury's eccentric orbit. The doppler shift of the solar spectrum at the D2 sodium resonance line (dotted blue line) is also shown at TAAs of 0°, 90°, 180°, and 270°. The white dotted line on Mercury, shows the location of the cold‐pole longitudes (longitudes with lower than average temperatures, Peplowski et al., 2014). Adapted from Cassidy et al. (2015, 2016). UVVS, UltraViolet and Visible Spectrometer.

This eccentric orbit, as well as the exospheric emission observed by the UltraViolet and Visible Spectrometer (UVVS) onboard MESSENGER is shown in Figure 2 at various TAAs (0° is at perihelion, 180° at aphelion). The varying radial distance from the Sun results in a varying solar irradiance, which is a factor of 2.3 greater at perihelion then at aphelion. The eccentricity also means that Mercury has a velocity component directed radially away from the Sun at 0°–180° TAA (it is moving away from the Sun and is therefore “outbound”) and has a velocity component directed toward the Sun at 180°–360° TAA (it is moving toward the Sun and is “inbound”). This changing radial velocity with respect to the Sun causes the solar spectrum to be Doppler shifted in Mercury's frame of reference. Furthermore, the sodium resonance lines lie in an absorption feature (a Fraunhofer line) of the solar spectrum. This absorption line is shifted to longer and shorter wavelengths on the outbound and inbound portions of its orbit, respectively (Figure 2). Therefore, the solar intensity varies with TAA at the resonance line. Combining this with a varying solar irradiance, results in a varying antisunward acceleration (or *g*‐value) that the neutral sodium experiences with TAA, peaking at 60° and 300°. This changing *g*‐value results in a varying exosphere due to the variation in sodium loss downtail (Potter et al., 2007). Cassidy et al. (2015) found a varying scale height and surface density of neutral sodium at Mercury's dayside which was anti‐correlated to the *g*‐value.

Even though there have been many ground and space measurements, the actual quantitative importance of all these sources and losses to the exosphere is ambiguous, largely due to the uncertainties of velocity distributions and sticking coefficients of the atom's interaction with the surface (McClintock et al., 2019). It has been suggested that PSD is the dominant source of the exosphere (McGrath et al., 1986). Impact vaporization has been suggested to contribute 1%–20% of the PSD source (Burger et al., 2010; Cremonese et al., 2005; Mouawad et al., 2011). Schmidt et al. (2012) estimated that to populate the lost sodium in the exospheric tail would require 2.7 × 10^24^ atoms/s from an impact vaporization source or up to 10^25^ atoms/s from PSD. For more information about the exosphere, we refer the reader to recent review chapters (Killen et al., 2019; McClintock et al., 2019).

Photoionization as a loss process to the exosphere has not been previously studied using ion and neutral measurements. Photoionization is the process by which neutral sodium is lost from the exosphere due to the ionization of sodium which is then picked up by the magnetospheric plasma. Using observations from UVVS and the Fast‐Imaging Plasma Spectrometer (FIPS) onboard MESSENGER, as well as the ground‐based THEMIS solar telescope, we investigate this process. In Section 2, we describe the methods used for analysis. We also present some estimates of the mass loss due to photoionization. In Section 3, we interpret the data and discuss what we have learnt from the results about Mercury's exosphere. In Section 4, we summarize our conclusions.

## 2 Observations and Photoionization‐Loss Calculations

In this study, we refer to neutral sodium as “sodium” and sodium ions as “Na^+^”. For this analysis, we used data from the following MESSENGER instrumentation: FIPS (Andrews et al., 2007) and UVVS (McClintock et al., 2007). We also use data reported by Milillo et al. (2021) from the THEMIS telescope (López‐Ariste et al., 2000). Background information about the instrumentation and the Mercury‐Solar‐Orbital (MSO) coordinate system can be found in the Online Supporting Material.

### 2.1 Plasma Measurements and Method

**Figure 3:**
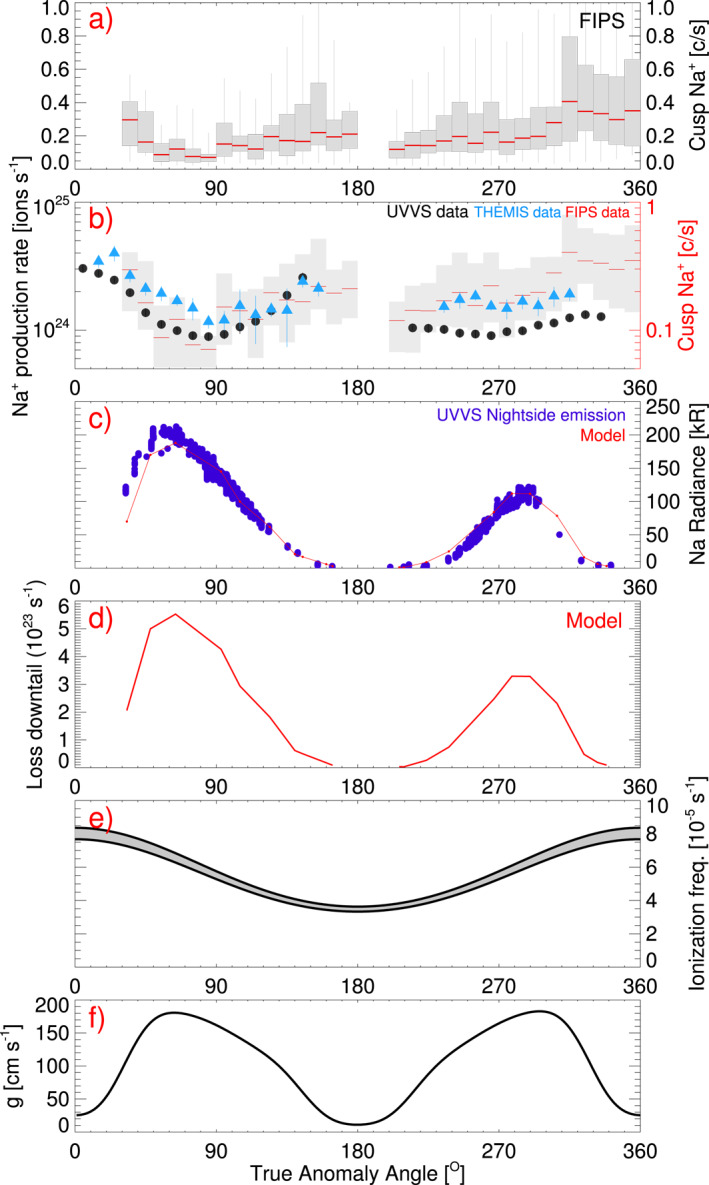
Sodium dependence on TAA. Panel (a) FIPS observed Na^+^ count rate in the cusp binned in 10° TAA bins (Section 2.1); (b) shows the total dayside Na^+^ production rate (i.e., the amount of ions produced, and therefore neutrals lost from the exosphere) calculated from UVVS measurements (black dots) and the THEMIS telescope (blue triangles). FIPS measurements of Na^+^ from panel (a) are also shown again on a log scale for comparison with neutral data. (c) UVVS observed nightside emission at midnight local time at 0.25 R_M_ above Mercury's surface (blue) and a model of nightside emission fit to the data (red); (d) modeled sodium loss downtail; (e) the ionization frequency of sodium, with the range representative of a quiet and active Sun. (f) the solar radiation acceleration of sodium at Mercury. FIPS, Fast‐Imaging Plasma Spectrometer; TTA, true anomaly angle; UVVS, UltraViolet and Visible Spectrometer.

During the MESSENGER mission the northern cusp was observed 2,780 times. We use the cusp crossings to analyze the FIPS‐measured Na^+^ count rate dependence with TAA. To account for any orbital or instrument effects that may affect our results, we prescribe certain requirements. First, we only use cusp crossings which measured at least 1 Na^+^ count. Second, since the spacecraft's altitude during cusp crossings varied during the MESSENGER mission, we only use cusp crossings with an average altitude of 0.15–0.25 R_M_ (a bin width of 244 km). Third, the FIPS instrument had a restricted field‐of‐view, since it did not have 4π steradian sampling of space. Therefore, we only include cusp crossings when the FIPS boresight vector pointed within 300°–30° (i.e., 300°–360° and 0°–30°) from the Y_MSO_ direction in the *Y*‐*Z*_MSO_ plane (0° and 90° being in the *Y*_MSO_ and *Z*_MSO_ directions, respectively). This results in observations of ions that have mirrored in the cusp and are largely traveling northwards in the dayside cusp (within a 90° FOV bin). This limits any effects on our results from ion anisotropy in the cusp. These bins were selected so as to retain as much of the data as possible. These requirements result in the analysis of 1,452 cusp crossings. We calculate the average Na^+^ count rate for each cusp crossing (by dividing the total sodium count by the total accumulation time made by FIPS, during a cusp crossing). Figure 3a presents the Na^+^ count rate binned in 10° TAA bins (where there were at least five cusp crossings). The gray boxes show the interquartile range (IQR), and the whiskers show 1.5 × IQR. The red lines show the median Na^+^ count rate within each TAA bin.

The Na^+^ count rate in the cusp is used as a proxy for the Na^+^ content for Mercury's magnetosphere. Estimating a global magnetospheric Na^+^ content is difficult due to the orbit of MESSENGER (the inclined orbit rotates in local time with TAA) and therefore it is difficult to disentangle the seasonal versus local time variations. If all the Na^+^ observations from MESSENGER are used, any dependence on TAA will be lost due to local time variations in the magnetosphere. This is seen in Raines et al. (2013; Figure 2c), where dayside and nightside observations are averaged in TAA using different regions (magnetosphere, magnetosheath etc.). This produces a result where magnetospheric local time asymmetries, such as the duskward drift of heavy ions in the magnetotail due to the curvature of the magnetic field (Delcourt et al., 2003), will be present and any TAA dependency will be lost.

We therefore focus on the northern magnetospheric cusp crossings as it is the only region in Mercury's magnetosphere that is regularly sampled throughout most of Mercury's year, making it a good proxy for the general trend of the Na^+^ dependency on TAA. We assume the Na^+^ is generated from photoionization. This is a reasonable assumption: charge‐exchange and electron‐impact ionization are negligible at Mercury due to the observed low‐densities. We also assume that there will be some “magnetospheric processing” of the cusp Na^+^ (after ionization), which energizes Na^+^ to the 1–10 keV/q energies observed by FIPS. Even with energization by the magnetosphere the Na^+^ still originate from photoionization of the sodium exosphere, and our method is justified in understanding sodium in the context of the exosphere.

### 2.2 Neutral Measurements

#### 2.2.1 Calculation of the Ion Production Rate From Photoionization of Sodium to Na^+^

Using UVVS measurements of the sodium density and scale height as they vary in TAA (Cassidy et al., 2015), we calculate an estimate of the mass loss to the exosphere due to photoionization. This is the estimated loss of sodium due to photoionization, consequently leading to the production of Na^+^. This estimate for the whole dayside exosphere is calculated by taking dayside observations from UVVS data at 2‐h local time bins at 0600–1800, using observations at low to mid‐latitudes (±45°), and tangent altitudes less than 400 km for the whole MESSENGER mission. The data is binned in 10° TAA bins (with at least three measurements per bin). The measured density and scale height are used to calculate the number of sodium atoms in the local time‐latitude volume. The sodium content of the dayside exosphere is then multiplied by the photoionization frequency to calculate the Na^+^ production rate. We assume the exosphere is optically thin, meaning the photoionization rate is not attenuated with decreasing altitude and remains constant with solar zenith angle and altitude. The optical depth *τ*, can be calculated using:

(1) $$\tau =\sec\;\chi \underset{s}{\sum }n_{s}\left(z\right)\sigma_{s}^{a}\left(\lambda \right)H_{s}$$

where *χ* is the solar zenith angle, *z* is altitude, *n*_*s*_ is the neutral density, $\sigma_{s}^{a}$(λ) is the wavelength‐dependent absorption cross section of species *s*, and *H*_s_ is the scale height (Schunk & Nagy, 2000). By using the photoionization cross section (scaled to Mercury's orbital location) for monoatomic sodium from Huebner and Mukherjee (2015) and using an exponential decay approximation for the neutral density and a surface neutral density of 0.9 × 10^5^ cm^−3^ (for TAA = 150°, corresponding to the maximum measured density), the optical depth is ∼10^−5^. An optical depth equal to unity would mean that the photoionization peak was reached, so a value of 10^−5^ implies that the optically thin approximation is valid. This validates our calculation of the ion production by simply multiplying the ionization frequency by the neutral density.

Figure 3b shows the estimation of the loss due to photoionization (and therefore the Na^+^ production rate) as black dots. The Na^+^ production rate estimate from UVVS data does not include the mid‐to‐high latitude enhancements seen in ground‐based data sets. Therefore, we also make the same calculation from ground‐based observations using disk‐averaged THEMIS‐telescope data reduced and analyzed by Mangano et al. (2015) and recently reported by Milillo et al. (2021) which include high latitude regions. Results are shown as blue triangles in Figure 3b. The FIPS‐measured Na^+^ ion count rate are also shown in gray/red on a log‐scale (i.e., the data from Figure 3a) for a direct comparison of the three data sets. Details about the uncertainty estimation can be found in the Online Supporting Material.

#### 2.2.2 Nightside Emission and Downtail Loss

Nightside sodium emission observed by UVVS are shown in Figure 3c in blue. UVVS observations at midnight local time and at 0.25 R_M_ (610 km) altitude were binned in TAA. These measurements were taken when MESSENGER was southward of Mercury and the instrument was facing northward (i.e., “looking upward” toward the limb of the planet). The line‐of‐sight for these observations include Mercury's umbra (visible in Figure 2). The observed emission comes from sodium in the northern and southern flanks of the tail.

We also used a simple Monte Carlo model (Burger, 2021, described by; Burger et al., 2014 and used to study sodium by; Cassidy et al., 2015) to estimate the sodium loss downtail. The radiance estimated from the model is shown in red (Figure 3c), and the corresponding sodium loss downtail is shown in Figure 3d. We assumed a simplified exosphere source: a 1200 K Maxwell flux distribution centered on the subsolar point and falling off as the cosine of the solar zenith angle (for more information about the model, we refer the reader to the Appendices found in Cassidy et al., 2015). The supply rate was set to match the observed radiance (Figure 3c), which results in a constant supply rate of 6 × 10^25^ sodium atoms/s. This supply rate assumes that each sodium atom “sticks” when it returns to the surface. In reality atoms are re‐ejected and re‐desorbed multiple times, an atom can be re‐desorbed 20–40 times during its photoionization lifetime (roughly estimated by dividing the photoionization lifetime by a ballistic lifetime), so the net supply rate is much lower.

Although oversimplified, this model reproduces many features of the UVVS nightside sodium emission data (Figure 3c, model in red; data in blue), most importantly the inbound/outbound asymmetry in tail radiance. This observed emission is of the nightside loss due to radiation acceleration. The loss rate during the outbound leg is about twice that of the inbound (a sodium atom was counted as lost if it traveled more than 15 R_M_ away from Mercury's center). We found that this outbound/inbound loss ratio is not especially sensitive to the spatial distribution of ejected sodium, but it does change with energy distribution. More energetic sodium produces a smaller ratio while a less energetic source results in a larger ratio. For example, a 600 K source has a ratio of ∼5 and a 4000 K source has a ratio of ∼1.25. Similar values were described by Schmidt et al. (2012). This model does not reproduce the dawn/dusk asymmetries (Cassidy et al., 2015), latitudinal asymmetries measured by ground‐based observers, or the high‐energy component seen above the high‐altitude dayside (Cassidy et al., 2015) or distant tail (Schmidt et al., 2012).

## 3 Discussion: What Have We Learned From the Observations?

The results are shown in Figure 3. The Na^+^ count rate in the cusp (binned in TAA) is shown in Figure 3a. This is also shown in Figure 3b, alongside the estimated ion production rate at Mercury (i.e., the exospheric loss rate due to photoionization) using neutral measurements from the MESSENGER spacecraft and the Earth‐based THEMIS‐telescope. Figure 3c shows the nightside sodium emission at altitudes of 0.25 R_M_ observed by UVVS (blue) and estimated by a simple model (red). Figure 3d shows the estimated sodium loss downtail from this model. For reference, Figure 3e shows the ionization frequency of sodium; where the upper and lower values are for an active and quiet Sun respectively. Figure 3f presents the antisunward *g*‐value experienced by sodium at the exosphere.

Mercury's Na^+^ content (Figure 3a) has a large variation for cusp crossings with the same TAA which can be seen by the large IQR (gray boxes). This variation is mostly likely due to magnetospheric effects, where separate isolated orbits may have different magnetospheric activity (i.e., MESSENGER‐observed orbit‐to‐orbit variation). However, the general seasonal TAA‐trend of the Na^+^ content is affected by the profiles of the *g*‐value (3f) and photoionization frequency (3e). The conditions are more conducive for Na^+^ production at 0° (and 360°) TAA when the photoionization frequency is at its highest and when the *g*‐value is at its lowest. If the *g*‐value is lower, less neutral sodium is lost by radiation acceleration, meaning more sodium is available for photoionization. Therefore, at 0° and 360° TAA, increases in the Na^+^ count rate are observed. The *g*‐value is also at a minima at 180° TAA. However, the photoionization frequency is also at a minimum, so MESSENGER does not observe as large an increase in Na^+^ at 180° as at 0°. When there is a peak in *g*‐value, sodium loss due to acceleration is at its highest, therefore there is less sodium available for photoionization. There is a clear minimum in the Na^+^ at 60° when the *g*‐value is at its highest. Similarly, at 300° TAA the *g*‐value is also at a maximum, however the Na^+^ does not exhibit a clear depression because there is less sodium lost downtail.

### 3.1 Different “Effective” *g*‐Value Between Outbound and Inbound Parts of Mercury's Orbit

Even though the *g*‐value at rest with Mercury is equal at TAA's of 60° and 300°, we now explain why more sodium can be lost at 60° than 300°. Figure 2 shows that the solar spectrum is shifted redwards (to longer wavelengths) on outbound TAA's of 0°–180°. The solar intensity at the resonance line therefore increases (as it is now on the “blue” side of the Fraunhofer line) and the sodium atom is accelerated. As the sodium atom accelerates away from the Sun, the solar spectrum that the atom observes is shifted further redward and the solar intensity further increases. This feedback loop produces a self‐accelerating mechanism where the sodium is further accelerated.

On the inbound portion of Mercury's orbit (180°–360°), the solar spectrum is shifted bluewards (toward shorter wavelengths). The solar intensity at the resonance line increases as it now lies on the “red” side of the Fraunhofer line. As the sodium atom is accelerated, the solar spectrum experienced by the atom is redshifted (opposite to the initial bluewards shift) which means the solar intensity at the resonance line is decreased. Consequently, even though the nominal *g*‐value at rest with Mercury can be the same at 60° and 300° TAA, the amount of sodium lost downtail is different. Therefore, the *g*‐value shown in Figure 3f is the *g*‐value for sodium at rest with Mercury (the same at 60° and 30°), whereas the “effective” *g*‐value is the *g*‐value experienced by an atom moving downtail. This mechanism was first proposed by Smyth (1986) and discussed by Smyth and Marconi (1995) and Potter et al. (2007). Even though this mechanism has been discussed in previous publications, it still has consequences on the loss rate from photoionization. As more sodium is lost downtail at 60° than 300° (as seen in Figure 3d), it affects how much neutral sodium is left to be photoionized. Therefore, the measured Na^+^ content by FIPS is higher on the inbound leg (180°–360° TAA) in comparison to outbound (0°–180° TAA).

### 3.2 Photoionization: Estimated Exospheric Loss Rates

The estimated dayside loss rate to the exosphere due to photoionization is calculated using surface density and scale height measurements by UVVS (Cassidy et al., 2015), shown in Figure 3b (black dots), and the THEMIS telescope reported by Milillo et al. (2021), shown as blue triangles. The estimates generally reproduce the maximum Na^+^ count rate observed in the FIPS data (gray and red in Figure 3b) at 0° TAA. The estimations also match the trend measured by FIPS very well for 0°–180° TAA, with peaks at 0° and 180° and a minima at ∼70°–80°. During the inbound portion of Mercury's orbit (180°–360°), the Na^+^ production rate from UVVS shows lower values for the Na^+^ production rate when compared to the FIPS measurements and the estimation from THEMIS. The THEMIS estimation is larger than UVVS because it includes more of the disk due to its high‐latitude coverage (UVVS is limited to mid‐latitudes). This is also seen for 0°–180° where THEMIS consistently estimates higher Na + production rates. This shows the importance of including high‐latitude sodium measurements when investigating Mercury's sodium exosphere. Furthermore, Milillo et al. (2021) find that different regions of Mercury's dayside provide different exospheric sodium contents. Understanding spatial variations is important for future observations and missions, however here we provide a first estimate from data.

The values that such an estimation produces are also important. At its maximum the calculation estimates that exospheric loss values from photoionization alone can be as high as 4 × 10^24^ atoms/s at perihelion (∼0° TAA). This is an important result and shows that with such a loss rate, photoionization would result in the total removal of the sodium exosphere within ∼4 h (if other source and loss process were to be “switched off,” and the loss rate was kept constant), which is therefore significant. This calculation is made for a dayside sodium exospheric content of ∼4 × 10^28^ atoms, from UVVS estimates of sodium dayside surface density and scale height reported by Cassidy et al. (2015).

Furthermore, only <15% of this Na^+^ is expected to be recycled back to the surface (Leblanc et al., 2003). Leblanc and Johnson (2003) modeled the exosphere and found an exospheric loss rate due to photoionization of 3.5 × 10^23^ atoms/s, an order of magnitude less than our maximum estimate (4 × 10^24^ atoms/s), and less than our minimum of 9 × 10^23^ atoms/s (by a factor of 2.5). Similarly, Mura (2012) underestimate Na^+^ production with an estimated peak value of 8 × 10^23^ s^−1^ (calculated using values from their Table 2). They use larger ionization frequencies which means they underestimate the sodium content of the exosphere. Wurz et al. (2010) mention that 7.45% of the sodium produced by PSD is lost by photoionization, however they do not specify production rates so we cannot directly compare our results. However, it is clear that models of Mercury's exosphere underestimate the importance of photoionization as a loss process.

When compared to other estimates of mass loss, photoionization is significant depending on the season. In comparison, escape rates due to radiation acceleration have been estimated to be between 5 and 13 × 10^23^ atoms/s (McClintock et al., 2008; Potter & Killen 2008; Schmidt et al., 2010). Photoionization‐loss values are equal or higher depending on season. The peak escape rate estimated for radiation acceleration occurred at 69° TAA (Schmidt et al., 2010), which is when the loss rate from photoionization is at its lowest. This shows that photoionization is comparable to radiation acceleration as a loss process to the exosphere at 69° TAA and at other TAA's loss due to photoionization is larger.

## 4 Conclusions

We have completed the first analysis of MESSENGER (FIPS and UVVS) and THEMIS‐telescope observations to understand photoionization as a loss mechanism to Mercury's sodium exosphere. Variations in the Na^+^ that are observed within a single TAA bin are likely due to variation in magnetospheric activity which can vary the Na^+^ content (i.e., MESSENGER orbit‐to‐orbit variation). Here we investigated the long‐term variations in Na^+^ depending on Mercury's TAA (i.e., season‐to‐season variation). We conclude the following:

1. Na^+^ production varies with Mercury's orbit around the Sun
2. Na^+^ production is affected by the *g*‐value. If more neutral sodium is lost downtail due to radiation acceleration then there is less neutral sodium to be photoionized
3. There is more Na^+^ production on the inbound leg, which is observed in the MESSENGER plasma measurements. This is due to more neutral sodium which is lost downtail on the outbound leg of Mercury's orbit (0°–180° TAA) than on the inbound (180°–360° TAA). This is caused by a self‐accelerating mechanism (see Section 3.1) on the outbound leg as first proposed by Smyth (1986)
4. Calculations using UVVS and THEMIS‐telescope measurements of the sodium exosphere show that exospheric sodium loss rates of ∼10^24^ atoms s^−1^ from photoionization (with a peak value of 4 × 10^24^ atoms s^−1^) are significant when compared to other loss mechanisms. This value is higher than previous modeled photoionization loss estimates. Photoionization is comparable to radiation acceleration as a loss process to the exosphere at 69° TAA and larger at other TAA's
5. Despite planetary differences in heliocentric distance, space environment processes, and atmospheric content, the estimated loss of sodium due to photoionization at Mercury is similar to ion loss rates at Venus and Mars (10^24^–10^25^ s^−1^; Futaana et al., 2017; Ramstad et al., 2017)

Although we have focused on the large‐scale effects of radiation acceleration affecting photoionization loss, we do expect other effects to contribute to the variation of the sodium exosphere due to spatial or temporal or orbital variations. More accurate and reliable modeling will be the focus of a future paper, including high‐latitude enhancements in sodium that can be inferred from other UVVS observation geometries.

## Supporting information

Supporting Information S1

Data Set S1

## Data Availability

All MESSENGER data are available on the PDS (https://pds-ppi.igpp.ucla.edu/search/?t=Mercury&sc=Messenger&facet=SPACECRAFT_NAME&depth=1). THEMIS data are available online (http://themis.iaps.inaf.it). The data shown in Figure 3 are available for download in the online supporting material.
