# Supplementary material for: Photoionization Loss of Mercury's Sodium Exosphere: Seasonal Observations by MESSENGER and the THEMIS Telescope
Source: Geophys Res Lett. 2021 Apr 28;48(8):e2021GL092980. doi: 10.1029/2021GL092980 (PMC8243941; doi:10.1029/2021GL092980)
Supplement: Supplementary file 1 — Supporting Information S1 [file GRL-48-e2021GL092980-s001.docx]

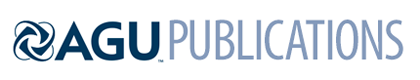


*Geophysical Research Letters*

Supporting Information for

**Photoionization Loss of Mercury’s Sodium Exosphere: seasonal observations by MESSENGER and the THEMIS telescope**

Jamie M. Jasinski^1,*^, Timothy A. Cassidy^2^, Jim M. Raines^3^, Anna Milillo^4^, Leonardo H. Regoli^5^, Ryan Dewey^3^, James A. Slavin^3^, Valeria Mangano^4^ and Neil Murphy^1^

1. NASA Jet Propulsion Laboratory, California Institute of Technology, Pasadena, CA, USA.

2. Laboratory of Atmospheric and Space Sciences, University of Colorado Boulder, CO, USA.

3. Dept. of Climate and Space Sciences and Engineering, University of Michigan, MI, USA.

4. INAF/IAPS, Italy.

5. Applied Physics Laboratory, John Hopkins University, MD, USA.

*Corresponding author: Jamie Jasinski (jasinski@jpl.nasa.gov)

**Contents of this file**

Text S1 – Instrumentation Information

Text S2 – Description of Dataset S1 “Data From Figure 3”

**Additional Supporting Information (Files uploaded separately)**

Dataset S1 – Data from Figure 3.

**Introduction**

Here we provide more detailed information about the instrumentation used in the analysis reported in the main article (Text S1). We also provide some additional information provided in the supplementary dataset (Dataset S1).

Text S1. – Instrumentation Information

We use Mercury Solar Orbital (MSO) coordinates, where X is in the planet‐Sun direction, −Y points toward the planetary orbital velocity vector direction, and Z completes the right‐hand set and points northward.

FIPS was a time-of-flight mass spectrometer which measured ions with an energy-per-charge of 0.46–13 keV/q, a mass-per-charge of 1–60 amu/q, and a maximum time resolution of ~9 s (2.6s accumulation and ~6s processing). The effective field-of-view was ~1.15π sr (~0.25π sr was blocked by the spacecraft sunshade). The signal-to-noise of heavy ions is improved by grouping heavy planetary ions into two groups: the Na^+^ group (m/e = 21–30 amu/e, including Na^+^, Mg^+^, Al+, and Si^+^). Na^+^ is considered the most dominant ion species observed at Mercury, and the we refer the reader to Raines et al., (2012) to this binning process.

UVVS was a telescope with a grating monochromator that scanned discrete narrow wavelength bands in order to detect exospheric emission. The sodium scan covered a wavelength range of 587.7-591.1 nm (with both the D1 and D2 emission lines – 589.8 and 589.1 nm respectively – falling at the center of the scan). The instrument’s sensitivity calibration was used to convert from counts per second to produce radiance for each spectral scan, and summed to calculate a total sodium radiance.

The THEMIS telescope is a solar telescope that is located in Tenerife, Spain (López-Ariste et al., 2000). It has a 0.9m primary mirror and a 15.04m focal length. THEMIS is used during the day to image Mercury’s Na exosphere. The MulTiRaies spectrometer with two separate cameras is used to observe the Na D1 and D2 lines, simultaneously. Data used here is from the D2 measurements between 2009 and 2013, which were reduced and processed by Mangano et al., (2015) and also recently reported by Milillo et al., (2021).

Surface density and scale height uncertainties calculated by Cassidy et al., (2015), along with the standard deviations are propagated and shown in Figure 3b as black lines which are smaller than the plot symbol (similarly to Cassidy et al., 2015, we do not include any systematic errors). Given that the indetermination due to seeing variability and to the intrinsic error in the calculation of the column densities is difficult to be evaluated for the THEMIS-telescope data, in the plot we used the standard deviation of the averaged column densities for each TAA interval (considered only when there are more than 5 observations) as error bars (blue lines). These values are generally higher than the pixel dispersion for each single image and, hence, can be considered as a valid upper limit.

Text S2. – Description of Dataset S1 “Data from Figure 3”.

The Excel spreadsheet provides the data shown in Figure 3, for the Na^+^ production estimate, the UVVS Na Nightside Emission, Ionization Frequency and the g-value.

References

López-Ariste, A., J. Rayrole, M. Semel First results from THEMIS spectropolarimetric mode A&AS, 142 (2000), pp. 137-148, 10.1051/aas:2000144

Mangano, V., Massetti, S., Milillo, A., Plainaki, C., Orsini, S., Rispoli, R., and Leblanc, F.: THEMIS Na exosphere observations of Mercury and their correlation with in-situ magnetic field measurements by MESSENGER, Planet. Space Sci., 115, 102–109, https://doi.org/10.1016/j.pss.2015.04.001, 2015.

Milillo, A., Mangano, V., Massetti, S., Mura, A., Plainaki, C., Alberti, T., et al. (2021). Exospheric Na distributions along the Mercury orbit with the THEMIS telescope. Icarus, 114179. https://doi.org/10.1016/j.icarus.2020.114179

Raines, J. M., et al. (2013), Distribution and compositional variations of plasma ions in Mercury's space environment: The first three Mercury years of MESSENGER observations, J. Geophys. Res. Space Physics, 118, 1604– 1619, doi:10.1029/2012JA018073.
